# Supplementary material for: COVID-19 and Pulmonary Embolism Outcomes among Hospitalized Patients in the United States: A Propensity-Matched Analysis of National Inpatient Sample
Source: Vaccines (Basel). 2022 Dec 8;10(12):2104. doi: 10.3390/vaccines10122104 (PMC9784895; doi:10.3390/vaccines10122104)

Diagnosis	ICD-10 code
Acute Pulmonary Embolism	I2602,I2609,I2692,I2693,I2694,I2699
Covid 19	U071, U00, U49, U50, U85, J1282
Mechanical ventilation	5A1945Z,5A1955Z,5A1935Z,5A09357,5A09457,5A09557
vasopressor	3E030XZ,3E033XZ,3E040XZ,3E043XZ,3E050XZ,3E053XZ,3E060XZ,3E063XZ
Sudden cardiac arrest	I46, I97
AKI and hemodialysis	N17, N990,5A1D70Z,5A1D90Z,5A1D80Z,5A1D00Z,5A1D60Z
Cardiogenic Shock	R570
Mechanical Circulatory Support	5A02110, 5A02210, 5A0211D, 02HA3RZ, 5A02116, 5A0221D, 5A1522F, 5A1522G, 5A1522H, 5A15A2F, 5A15A2G, 5A15A2H, 5A15223
CKD	N181, N182, N1830, N1831, N1832, N184,N185,N189
Pulmonary Circulation Disorder	Data Obtained from elixhauser comorbidity index
Chronic Pulmonary Disease	
Diabetes Uncomplicated	
Diabetes Complicated	
Hypothyroidism	
Peptic Ulcer Disease (excluding bleeding)	
Lymphoma	
Metastatic Cancer	
Solid Tumor Without Metastasis	
Rheumatoid Arthritis/Collagen Vascular	
Obesity	
Drug Abuse	
HTN	
PAD	
OSA	
Liver Disease	
Alcohol	
Smoking	F17, F172, F1720, F17200, F17201, F17203, F17208, F17209, F1721, F17210, F17211, F17213, F17218, F17219, F1722, F17220, F17221, F17223, F17228, F17229, F1729, F17290, F17291, F17293, F17298, F17299, Z87891
Hx of PCI	Z986, Z9861, Z9862
Hx of CABG	Z951
Previous MI	I252
CAD	I2510, I25111, I25118, I25119, I252, I253, I254, I2541, I2542, I255, I256, I257, I2570, I25700, I25701, I25708, I25709, I2571, I25710, I25711, I25718, I25719, I2572, I25720, I25721, I25728, I25729, I2573, I25730, I25731, I25738, I25739, I2575, I25750, I25751, I25758, I25759, I2576, I25760, I25761, I25768, I25769,

	I2579, I25790, I25791, I25798, I25799, I258, I2581, I25810, I25811, I25812, I2582, I2583, I2584, I2589, I259

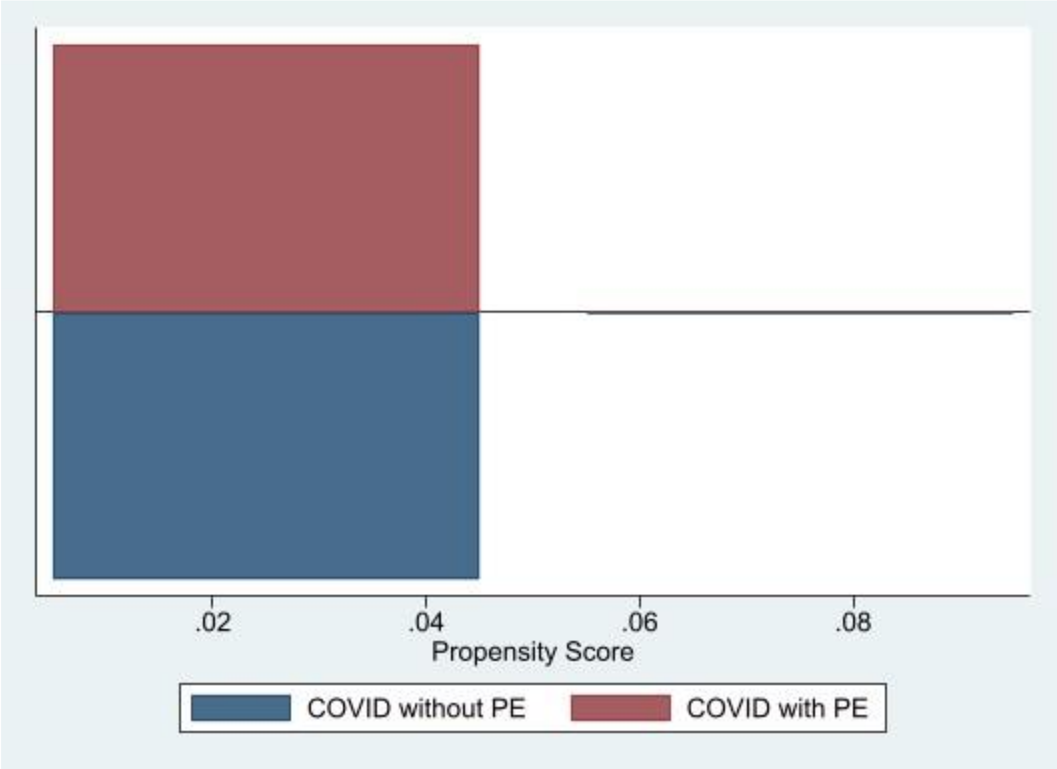

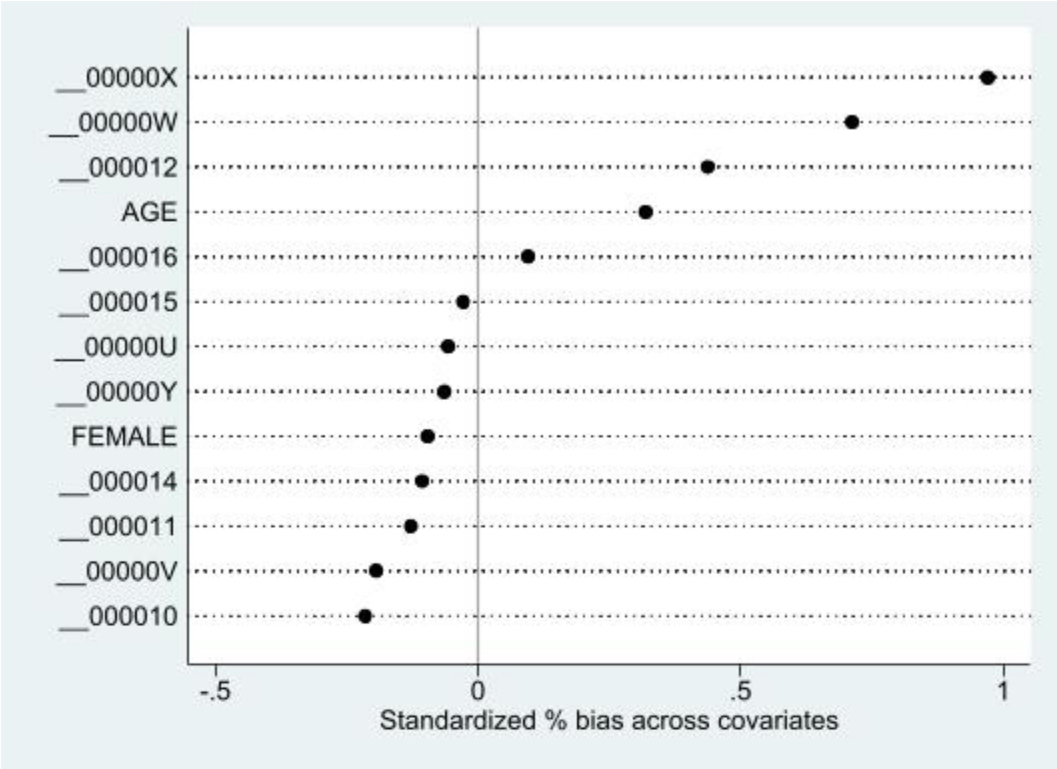

Supplement: Supplementary file 1 [file vaccines-10-02104-s001.zip › vaccines-2036763-supplementary.pdf]
